# The NLRP3 Inflammasome in Age-Related Cerebral Small Vessel Disease Manifestations: Untying the Innate Immune Response Connection

**DOI:** 10.3390/life13010216

**Published:** 2023-01-12

**Authors:** Che Mohd Nasril Che Mohd Nassir, Thenmoly Damodaran, Nurul Iman Ismail, Sabarisah Hashim, Usman Jaffer, Hafizah Abdul Hamid, Muhammad Zulfadli Mehat, Anwar Norazit, Muzaimi Mustapha

**Affiliations:** 1 Faculty of Applied Sciences, Universiti Teknologi MARA (UiTM), Perak Branch, Tapah Campus, Tapah Road 35400, Perak, Malaysia; 2 Neuro Psychological and Islamic Research and Consultancy Pty. Ltd., 32 Jane Avenue Rylands Est., Cape Town 7764, South Africa; 3 Department of Biomedical Science, Faculty of Dentistry, AIMST University, Bedong 08100, Kedah, Malaysia; 4 Department of Neurosciences, School of Medical Sciences, Universiti Sains Malaysia, Kubang Kerian 16150, Kelantan, Malaysia; 5 Kulliyyah of Islamic Revealed Knowledge and Human Sciences, International Islamic University Malaysia, Kuala Lumpur 50728, Malaysia; 6 Department of Human Anatomy, Faculty of Medicine and Health Sciences, Universiti Putra Malaysia, Serdang 43400, Selangor, Malaysia; 7 Department of Biomedical Science, Faculty of Medicine, Universiti Malaya, Kuala Lumpur 50603, Malaysia; 8 Hospital Universiti Sains Malaysia, Jalan Raja Perempuan Zainab II, Kubang Kerian 16150, Kelantan, Malaysia

**Keywords:** cerebral small vessel disease, NLRP3 inflammasome, cerebral ischemia, therapeutics, aging

## Abstract

In this narrative review, we present the evidence on nucleotide-binding and oligomerization (NOD) domain-like receptor (NLR) family pyrin domain (PYD)-containing 3 (NLRP3) inflammasome activation for its putative roles in the elusive pathomechanism of aging-related cerebral small vessel disease (CSVD). Although NLRP3 inflammasome-interleukin (IL)-1β has been implicated in the pathophysiology of coronary artery disease, its roles in cerebral arteriothrombotic micro-circulation disease such as CSVD remains unexplored. Here, we elaborate on the current manifestations of CSVD and its’ complex pathogenesis and relate the array of activators and aberrant activation involving NLRP3 inflammasome with this condition. These neuroinflammatory insights would expand on our current understanding of CSVD clinical (and subclinical) heterogenous manifestations whilst highlighting plausible NLRP3-linked therapeutic targets.

## 1. Introduction

A recent report from the Center for Disease Control and Prevention has indicated that 1 in 6 mortalities from non-communicable cardio-cerebrovascular disease is due to stroke [1]. The ischemic stroke (i.e., blockage of cerebral blood flow) represents 87% of all stroke cases [2]. Ischemic stroke is more prevalent with aging and is a leading cause of consequential long-term disability and reduced mobility [2]. Of note, cerebral small vessel disease (CSVD) accounts for about one-fifth (20%) of all strokes, 65% of ischemic stroke subtypes, and is the most common source of age-related cognitive decline and dementia and/or vascular dementia [3,4]. However, to date, there is limited knowledge of preventive measures to arrest its onset and progression despite several reported trials and interventions to modify the course of CSVD [5].

The research pertaining to CSVD has gained much interest worldwide given that CSVD may lead to various vascular-related brain injuries through multiple mechanisms that could be synergistic and/or cumulative. In the presence of cardio-cerebrovascular disease risk factors such as aging, hypertension, type-2 diabetes mellitus (T2DM), and cerebral amyloid angiopathy (CAA) mediated by vascular deposition of β-amyloid, the common underlying pathophysiological mechanisms of CSVD are primarily linked to thrombo-inflammation and arteriolosclerosis of penetrating cerebral micro-vessels (50–400 μm in diameter) [6]. Moreover, due to the largely elusive and likely overlapping pathomechanisms of CSVD, there is limited data available for its therapeutic strategy. Nevertheless, various anti-inflammatory agents have been investigated to explore the prospective treatment for inflamed cerebral ischemic tissue [7]. Corticosteroids have also been proposed as a distinct pluripotent immuno-suppressive agent that can be beneficial for ischemic stroke therapy [8]. However, the chronic administration of glucocorticoids to stroke patients leads to an increased prevalence of non-communicable to communicable diseases such as pneumonia [9]. Therefore, exploring new potential therapies targeting specific but prominent pro-inflammatory signals in ischemic stroke and CSVD is a timely translational effort.

Inflammasomes are protein complexes that activate caspase-1 and control the maturation of interleukin-1 (IL-1), a potent pro-inflammatory cytokine, which is triggered by a variety of endogenous and exogenous signals [10]. Over the past decades, extensive research has been done to study the inflammasome complex and its interrelationships with cardio-cerebrovascular diseases. The nucleotide-binding and oligomerization (NOD) domain-like receptor (NLR) family pyrin domain (PYD)-containing 3 (NLRP3) inflammasome is one of the most comprehensively investigated inflammasomes. Multiple studies have reported that the activation and expression of NLRP3 inflammasome fosters the progression of athero-, arterio-, and/or arteriolosclerosis lesions, hence increasing the risk for ischemic stroke [11,12,13]. Moreover, the activation of the NLRP3 inflammasome causes exacerbation of ischemic stroke and full-blown stroke, whilst the inhibition of NLRP3 inflammasome may ameliorate the clinical symptoms and diagnosis. Therefore, the objective of this narrative review is to highlight the current neuroimaging manifestations of CSVD and its complex pathogenesis, as well as to connect the array of activators and aberrant activation that may implicate the NLRP3 inflammasome in this condition. This would expand our current knowledge of the NLRP3 inflammasome as well as potential therapeutic strategies for CSVD as arguably the most prevalent age-related cerebrovascular disease.

## 2. The NLRP3 Inflammasome: Structure, Activation, and Role in Cardio-Cerebrovascular Diseases

An inflammasome is a multiple protein complex, which comprised of sensor proteins such as pattern recognition receptors (PRRs), an effector protein (i.e., caspase-1 in canonical inflammasome, and caspase-4,5,11 in non-canonical inflammasome), and an adaptor protein (i.e., apoptosis-associated speck-like protein, ASC—containing caspase activation and recruitment domain, CARD). An inflammasome modulates the innate immune signaling where PRRs respond to pathogen-associated molecular patterns (PAMPs) and/or damage-associated molecular patterns (DAMPs), which results in the activation and accumulation of caspase-1 that cleaves pro-interleukin (IL)-1β and 18 to their active forms. Activated pro-inflammatory cytokines (i.e., IL-1β) modulate inflammation in a series of disorders, including chronic inflammatory disease and neurodegenerative disease [14].

### 2.1. NLRP3 Inflammasome: Structure, Activation, and Role in Cardio-Cerebrovascular Diseases

As applied to PRRs, inflammasomes can be classified as interferon (IFN)-c inducible protein 16 (IFI16), absent in melanoma 2 (AIM2), and numerous NLR subsets [15]. Furthermore, PRRs can be sub-categorized into two main groups based on their cellular localization: (1) some toll-like receptors (TLRs) that are located in the plasma membrane which help to recognize extracellular DAMPs and PAMPs, (2) AIM2-like receptors and NLRs that are found inside the cell and are responsible for detecting intracellular DAMPs and PAMPs, and (3) subcellular interferon gamma inducible protein 16 (IFI16) [16].

Structurally, the NLRs are made up of three main components. The first component is the middle nucleotide-binding and oligomerization domain (NACHT) that exists in all NLRs, which contain adenosine 5’-triphosphatase (ATPase) activity 110, which is crucial for NLRP3 oligomerization [17,18]. The second is C-terminal, which inhibits the function of the NLR protein when leucine-rich repeats (LRRs) are inactivated or in a resting state and adjusts the conformation following the recognition of stimuli to eliminate the inhibitory effect on the NLR protein [19]. The third component is the N-terminal effector domain made up of either pyrin, CARD, or the baculoviral inhibitor of apoptosis protein repeat (BIR) domain before the NACHT domain [20]. Moreover, the NLRs can be further sub-classified into two groups based on the N-terminal domain. Firstly, the NLR sub-family C (NLRC) that involves CARD, and secondly, the NLRP containing pyrin [16]. These N-terminal domains instigate a cascade of specific downstream signaling via certain homotypic protein interactions.

**Figure 1:**
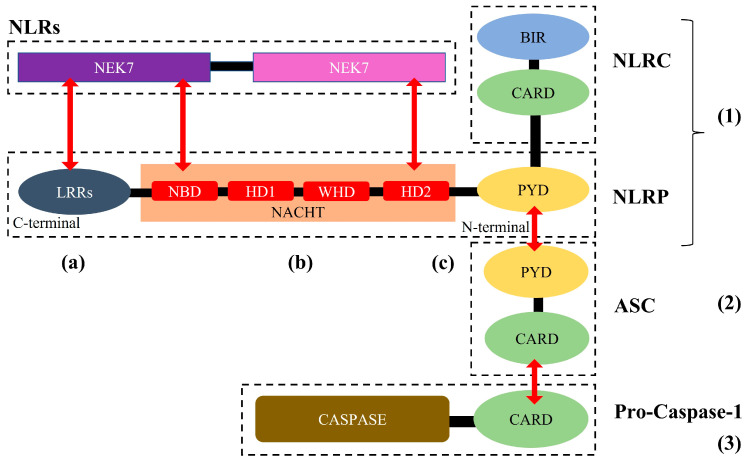
Structure of nucleotide-binding and oligomerization (NOD) domain-like receptor (NLR) family pyrin domain (PYD)-containing 3 (NLRP3) inflammasome. NLRs comprised of (**a**) C-terminal leucine-rich repeats (LRRs); (**b**) the central nucleotide-binding and oligomerization domain (NACHT)—made up of nucleotide-binding domain, NBD, helical domain, HD–1 and 2, and winged-helix domain, WHD; and (**c**) N-terminal pyrin domain (PYD). When the N-terminal part with PYD and caspase activation and recruitment domain (CARD)—the structure is called NLRP, but when the N-terminal consists of PYD and baculoviral inhibitor of apoptosis protein repeat (BIR)—the structure is called NLRC. The NLRP3 inflammasome is made-up of three components (or domains), which are the (1) LRRs-NACHT-PYD, (2) PYCARD (or PYD-CARD) or known as apoptosis-associated speck-like protein (ASC), and (3) Pro-caspase-1 (CARD + Caspase). The three-component merged through interaction of PYD-PYD and CARD-CARD, hence forming NLRP3 inflammasome complex. Moreover, NIMA-related kinase 7 (NEK7) is another part of NLRP3 inflammasome that is related to ROS-induced priming.

A well-established inflammasome complex that is encoded by the *nlrp3* gene is the NLRP3 inflammasome. This inflammasome is made up of three components: the innate immune receptor, i.e., a NLRP3 scaffold that contains three domains including the NACHT domain (which is made up of nucleotide-binding domain, NBD, helical domain, HD—1 and 2, and winged-helix domain, WHD), C-terminal LRRs domain, and N-terminal PYD effector domain [21]. The next component includes cysteine protease precursor pro-caspase (made up of caspase domain and CARD) [10,16]. Finally, the ASC is made up of PYCARD (i.e., N-terminal PYD and C-terminal CARD), which activates caspase-1 (Figure 1). Moreover, the NLRP3 inflammasome is primarily located in immune cells such as antigen-presenting cells macrophages, neutrophils, monocytes, and dendritic cells [16]. Furthermore, in the brain, the activated NLRP3 inflammasome is primarily derived from microglia cells whilst the activated ASC is derived from neuronal cells [22].

### 2.2. Activation of the NLRP3 Inflammasome

Distinct from other PRRs (i.e., TLR, C-type lectin receptors [CLR], and RIG-I-like receptors [RLR]), the primary amount of the NLRP3 inflammasome in immune cells is limited [23]. The pyrin domain of ASC is the site where NLRP3 can adhere to in order to recruit pro-caspase-1 by CARD–CARD interactions. The recruitment of pro-caspase-1 leads to the liberation of active catalytic p10 and p20 caspase-1 fragments, enabling the cleaving of inflammatory cytokine, i.e., pro-IL-1β and pro-IL-18 to their active states [19].

**Figure 2:**
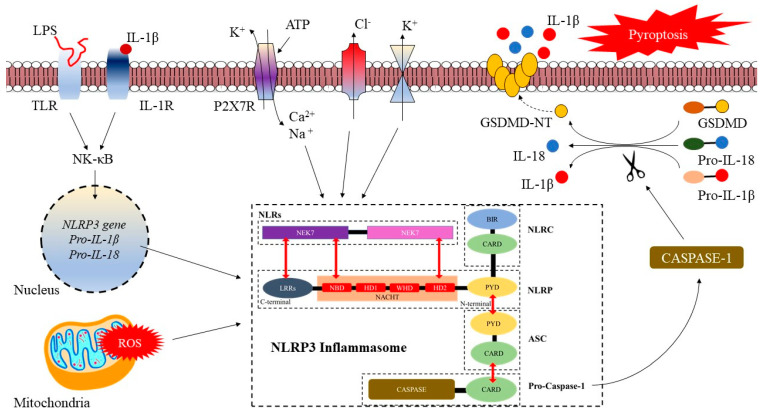
Mechanism of nucleotide-binding and oligomerization (NOD) domain-like receptor (NLR) family pyrin domain (PYD)-containing 3 (NLRP3) inflammasome activation. Upon certain cellular stress and/or elevated thrombo-inflammation, the increase in oxidative stress induced the over-activation of adenosine triphosphate (ATP), activates the purinergic ligand-gated ion channel 7 receptor (P2X7R), hence elevating calcium ion (Ca^2+^) and sodium ions (Na^+^) influx, and potassium ions (K^+^) efflux. Following that is the increased production of reactive oxygen species (ROS). The elevated production of ROS is also due to mitochondria dysfunction mediated by oxidative stress. Besides, following physiological stress, an increased stimulation of toll-like receptors (TLRs) by lipopolysaccharide (LPS) and interleukin (IL)–1 receptor (IL-1R) by extracellular IL-1β induced the activation of nuclear factor kappa-light-chain enhancer of activated B cells (NF-κB) that subsequently elevated the gene expression of NLRP3, pro-IL-18, and pro-IL-1β. The activated NLRP3 inflammasome mediates the pro-caspase self-cleavage into caspase 1. Caspase-1 lyses pro-IL-1β, pro-IL-18, and gasdermin-D-mediated cell death (GSDMD) into their active form, leading to pyroptosis or cell-death.

The activation of the NLRP3 inflammasome consists of a two-step process: priming and inflammasome activation. Priming refers to the signaling of inflammasome activation that is prompted by TLRs/nuclear factor kappa-light-chain enhancer of the activated B cells (NF-κB) pathway [24]. The NF-κB pathway can be activated by either TLRs that sense DAMPs and PAMPs, or cytokines (i.e., tumor necrosis factor α, TNF-α), or physiological stress that can result in an overexpression of NLRP3, pro-IL-18, and pro-IL-1β [25]. Besides that, the NLRP3 activation threshold is modulated by both post-transcriptional and translational activation of the *nlrp3* gene [17]. The activation of the NLRP3 inflammasome (particularly in macrophages) is dependent on *nlrp3* gene expression [26]. However, the NLRP3 remains inactive following the priming, although it is more reactive to any danger signals [27] (Figure 2).

The second step is the activation or trigger, whereby under certain signals or conditions (i.e., oxidative stress, thrombo-inflammation, or infection), the NLR will be activated and associated with ASC and pro-caspase-1 in a cascade response to form a complex structure. Synonymously, this complex mediates the pro-caspase-1 self-cleavage into caspase-1. Caspase-1 will then cleave pro-IL-1β, pro-IL-18, and the pore-forming molecule gasdermin-D (GSDMD) into their active forms [28,29]. Moreover, several conditions trigger or activate the NLRP3 inflammasome, and such conditions include the most crucial one, i.e., potassium (K^+^) efflux, an increase of reactive oxygen species (ROS) induced by PAMPs and DAMPs, and the release of cathepsin B by lysosomes [30]. Additionally, mitochondrial dysfunction, calcium (Ca^2+^) influx, chloride (Cl^-^) efflux, and sodium (Na^+^) influx also play an important role in the second signals for the activation of NLRP3 inflammasome [18,19] (Figure 2).

Previous reports have revealed that the activation of purinergic ligand-gated ion channel 7 receptor (P2X7R) plays a key role in neurodegenerative disease. Increased activation of P2X7R signaling influences the pro-inflammatory cytokines (i.e., IL-18, IL-1b, and TNF-α) and ROS (i.e., hydrogen peroxide) [31], which induced NF-kB signaling, and hence activates the NLRP3 inflammasome and subsequent cellular death [31]. The dying cells may increase the production and release of ATP and degenerative cycle. Moreover, P2X7R can mediate the over-production of intracellular ATP, hence increasing the upregulation of purinergic signaling and inflammation [32]. Following that is the elevation of Ca^2+^_,_ Na^+^ influx, and K^+^ efflux, which increases the production of ROS. The elevated production of ROS is also due to mitochondria dysfunction mediated by oxidative stress [33].

### 2.3. The Role of NLRP3 Inflammasome in Cerebrovascular Diseases

An increased expression of pro-inflammatory cytokines such as IL-1β has been widely studied and linked to cerebral infarction with the NLRP3 inflammasome and its inflammatory pathways (including caspase-1 and IL-1β) [34,35,36]. Moreover, IL-1β is mainly activated by the IL-1β converting enzyme called caspase-1 [37], which causes the elevation of cerebral infarct size by instigating the infiltration of neutrophil, and adherence at the infarct locus [34]. However, the infarct size and volume, as well as the neurological deficits caused by middle cerebral artery occlusion, were reported to be ameliorated after caspase-1 and IL-1β were knocked out or inhibited [38,39]. Furthermore, previous studies have linked the increased IL-1β expression to early brain aneurysm in pre-clinical mice models, and that IL-1β gene knockout diminishes the occurrence of cerebral vascular ballooning [40].

Previous reports also indicated that the activation of the NLRP3 inflammasome and its pathway elevate the blood–brain barrier (BBB) permeability, microglial aggregation, and neuronal cell death [41]. This may indicate that the NLRP3 inflammasome could interrupt the integrity of the neuro-glio-vascular unit system dynamics [42], thereby influencing cerebral interstitial fluidopathy (i.e., aberrant glymphatic clearance) [43,44] and age-related low-grade inflammation (i.e., inflammaging) [45]. Interestingly, recent studies have highlighted the inhibition of the NLRP3 inflammasome and its pathway mitigates the cerebral and cerebellar infarction in terms of size and volume, secondary brain injury and/or inflammation following cerebral hemorrhage, preserving the integrity and permeability of the BBB, and helping against neurological function loss and deficits [46,47,48].

The interrelation of the NLRP3 inflammasome and other cerebrovascular and/or neurodegenerative diseases such as Alzheimer’s disease (AD) has also been investigated. Neuroinflammation has been reported to cause the progression of AD, and elevated activation of pro-inflammatory cytokines such as IL-1β was detected in serum, cerebrospinal fluid (CSF), and brain parenchyma of patients with AD, whereby IL-1β caused a neurotoxic reaction against the neuro-glio-vascular unit [49,50,51]. Furthermore, IL-1β has also been found in patients with Parkinson’s disease (PD) [52,53]. The accumulation of α-synuclein (or Lewy bodies) that impedes the release of neurotransmitters has been identified as a general indicator of PD. Lewy bodies may activate the NLRP3 inflammasome via both mitochondrial dysfunction and TLRs [54]. The inhibition of IL-1β and the NLRP3 inflammasome has emerged as a new target of interest for the prevention and treatment of AD and PD.

Hence, there are sufficient plausible leads to posit that the NLRP3 inflammasome plays an important role in the pathophysiology of cerebrovascular disease, and the modulation (i.e., activation or blocking) of the NLRP3 inflammasome or caspase-1 may influence IL-1β synthesis and offer therapeutic avenues for cerebrovascular diseases, notably CSVD.

### 2.4. The Mutation of nlrp3 Gene against NLRP3 Inflammasome Pathway

As described previously, the activation of the NLRP3 inflammasome is dependent on *nlrp3* gene expression [26]. Where the priming step of NLRP3 inflammasome activation is proportionate to the increment of *nlrp3* gene expression [55]. Furthermore, an increased pathogenic stimulus may influence the activation of the NLRP3 inflammasome following *nlrp3* gene expression upregulation. A recent study has shown that *nlrp3* gene expression was upregulated through the NF-κB pathways following the interaction of TLR with its various agonists, such as Poly (I:C) and Pam3CysK4 [26].

Pre-clinical studies have shown that the ablation (i.e., genetic deletion) or mutation of the *nlrp3* gene potentially mitigates various age-related degenerative changes (i.e., bone loss, cardiac aging, ovarian aging, and insulin sensitivity with glycemic control) by interfering in the NLRP3 inflammasome activation pathways [56,57,58,59]. Moreover, Osario and colleagues, in their animal study, have shown that genetic modification and/or deletion of NF-κB signaling may help in the prevention of age-associated disorders [60]. Besides that, the deletion of the *nlrp3* gene has been shown to inhibit IGF-1 signaling and PI3K/AKT/mTOR (i.e., the intracellular energy sensor—associated with increase cellular autophagy), and other stressors (i.e., hypercaloric diet), hence improved various organism lifespan and aging [57,61,62].

## 3. Cerebral Small Vessel Disease (CSVD) and NLRP3 Inflammasome

### 3.1. CSVD: Pathophysiological Mechanisms

The pathophysiological basis of CSVD involves changes in the structure and function of cerebral microvasculature that penetrates in deep subcortical regions [63], such as arteries (chiefly the middle cerebral artery tributaries) and/or arterioles as well as lipohyalinosis, microthrombosis, necrosis, and fibrinolysis [64]. CSVD is common with aging and is frequently discovered as an incidental finding after neuroimaging. It is often overlooked by physicians due to its covert nature (i.e., asymptomatic). The neuroimaging manifestation of CSVD includes white matter hyperintensities (WMHs) of presumed vascular origins, enlarged perivascular spaces (ePVS), lacunar infarcts, cerebral microbleed (CMBs), and cortical microinfarcts. Alarmingly, these manifestations account for approximately 25% of the total global cases of ischemic stroke, and over 70% of vascular dementias [3].

Common cardio-cerebrovascular risk factors such as aging, hypertension, T2DM, smoking, and dyslipidemia increase the chances of developing a pathological change in the arteries and/or arteriole leading to vessel occlusion, hence arterio- and/or arteriolosclerosis [64]. This is also accompanied by the proliferation of connective tissue and ePVS, which in turn leads to the loss of vascular contractility that results in vascular sclerosis [65]. Endothelial dysfunction caused by a disrupted BBB permeability, CAA, and the recently reported formation and accumulation of cellular-derived microparticles are also factors in the etiopathogenesis of CSVD [66]. These changes lead to cerebral blood flow (cBF) disorder or hypoperfusion, which is correlated with atypical self-regulation and disrupts vascular wall permeability that causes multi-focal ischemia [67].

An increase in systemic inflammatory agents such as IL-1β, IL-6, and C-reactive protein (CRP) plays the most important roles in the genesis of neuroinflammation in CSVD and ischemic stroke [67]. The heightened pro-inflammatory agents alongside endothelial dysfunction (i.e., due to the formation and accumulation of cell-derived microparticles and disrupted purinergic signaling) may further aggravate endothelial injury. For example, microthrombi and/or microparticles may aggregate on the endothelial surface, worsening BBB permeability and leading to microvascular bleeding [68]. Furthermore, inflammation may disrupt cell–cell interactions, exacerbating the cellular injury that results in luminal narrowing, reduced cBF, hypoxia, neuronal cell death, and parenchyma damage [68].

Moreover, reduced cBF leads to excitotoxicity and energy failure, hence causing the increment of intracellular Na^+^ and Ca^2+^. Increased Ca^2+^ will then lead to mitochondrial dysfunction, which increases free radicals and ROS that further elevate the production and expression of inflammatory cytokines such as IL-1, IL-1β, and IL-6 [11]. This cascade increases microglial activation and leukocyte infiltration, resulting in widespread neuroinflammation and cell death. Furthermore, an increase in intracellular Na^+^ causes peri-infarct depolarization and increases K^+^ efflux. Consequently, these lead to cerebral ischemia, including CSVD manifestation [69].

### 3.2. The Hypothetical Link between NLRP3 Inflammasome and Manifestations of CSVD

**Figure 3:**
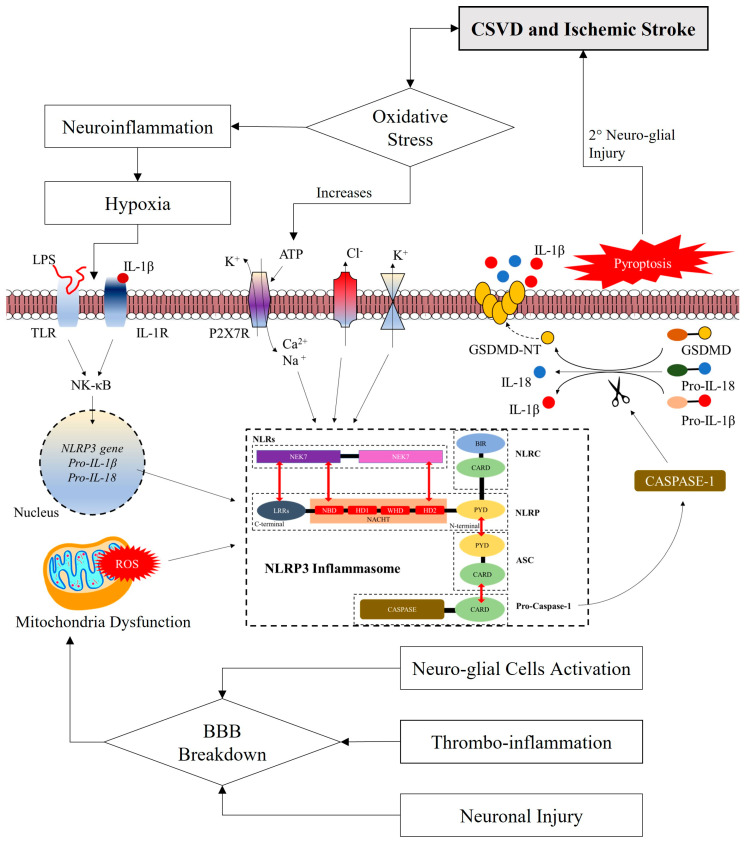
Hypothetical pathophysiological mechanisms to implicate NLRP3 inflammasome in CSVD. Blood–brain barrier (BBB) breakdown caused by elevated thrombo-inflammation, neuronal injury, and activation of neuro-glial cells mediates the mitochondrial dysfunction leading to increased production of reactive oxygen species (ROS). ROS activated the NLRP3 inflammasome leading to pyroptosis and secondary neuronal injury that may lead to the development and progression of cerebral small vessel disease (CSVD). Besides, cellular oxidative stress also causes hypoxia-mediated nuclear factor kappa-light-chain enhancer of activated B cells (NF-κB) pathway activation that subsequently led to NLRP3 inflammasome activation.

Following parenchyma injury, sequences of pathological changes that ensue could eventually elicit the activation of the NLRP3 inflammasome. The activated NLRP3 inflammasome may further worsen the parenchyma injury through a cascade of inflammatory signaling. As aforementioned, the NLRP3 inflammasome is crucial in the genesis of athero-, arterio-, and arteriolosclerosis and increases the likelihood of CSVD and ischemic stroke. Thus, here we hypothesize plausible pathophysiological mechanisms that underlie the NLRP3 inflammasome-linked CSVD through the NLRP3-mediated neuro-thrombo-inflammation, its influence on disease progression and potential therapeutic target(s) (see Figure 3).

### 3.3. Neuro-Thrombo-Inflammation: The Involvement of NLRP3 Inflammasome

Neuro-thrombo-inflammation is currently gaining attention in CSVD research. It is a complex innate immune-inflammatory cascade in the brain modulated by pro-inflammatory cytokine and pro-oxidative ROS (see Figure 3). These modulators mainly originate from the neuro-glio-vascular unit including neurons, microglia, astrocytes, oligodendrocytes, peripheral immune cells, and microvascular endothelial cells [70,71].

Following systemic stress and cellular insults, microglia are among the first to be activated and emigrate to the location of insult to mediate the innate immune response, which results in the release of inflammatory factors, promoting pathogen invasion and neuronal cell death [70]. The inflammatory factors (or mediators) in turn mediate the proliferation of astrocytes and magnify the inflammatory cascade by producing more inflammatory factors such as cytokines [70,71]. Furthermore, following cellular stress and insult, the production of cellular debris (referred to as microparticles or ectosomes) also occurs. The consequence of cellular inflammation is an increase in the proteolytic breakdown of the cytoskeleton, which will eventually cause a direct plasma membrane deformation and membrane phospholipid bilayer blebs, resulting in the formation of microparticles (0.1 to 1 μm in diameter) that are released from the cell surface [72]. The systemic accumulation of cellular-derived microparticles may cause the formation of microthrombus, hence leading to CSVD [55]. Taken together, neuro-thrombo-inflammation may cause the aggregation of immune cells, tissue or parenchyma damage, and/or cell death (apoptosis) [73].

As previously stated, the pivotal role of the NLRP3 inflammasome is the production of pro-inflammatory agents such as IL-1β that are involved in neuroinflammation. IL-1β is mainly produced by microglia and manifested in multiple conditions, including AD [74], PD [75], stroke [76], post-operative cognitive disorder [77], and depression [78]. In this case, the NLRP3 inflammasome influences the proliferation of astrocytes and the accumulation of microglia [79]. Moreover, previous studies have also reported that, following CSVD or cerebral ischemia, the NLRP3 inflammasome could possibly be activated in microglia cells through the activation of a purinergic signaling cascade by P2X7R and TLRs that recognize PAMPs and DAMPs [70,80].

Furthermore, previous pre-clinical studies have suggested that following ischemia, the increased expression of IL-1β after NLRP3 inflammasome activation may influence the permeability of cerebral microvasculature (including endothelial cells) and thus downregulate the expression of specific structures in the BBB (i.e., occluding tight junction proteins and zona occludens-1) [81,82,83]. Recent studies have also supported the activation of the NLRP3 inflammasome and NLRP3 inflammasome-mediated pyroptosis following ischemia, which modulate the polarity and distribution of aquaporin-4 in the infarct area [84], leading to an increased BBB permeability [85]. However, further studies on the exact mechanism are warranted [86]. Additionally, the recruited neutrophils in advanced or age-related CSVD may contribute to these BBB damages and could inflict a global cerebral ischemia and/or bleeding [87].

Therefore, it appears that neuro-thrombo-inflammation and the NLRP3 inflammasome are jointly implicated in the pathophysiological mechanism of CSVD and ischemic stroke or cerebrovascular disease in general. At the outset of cerebral ischemia, the neuro-thrombo-inflammation may disrupt the integrity of the BBB, thus promoting neuronal degeneration, axonal loss, neuronal cell death, and parenchymal damage. Pre-clinical animal models have also shown that elevated pro-inflammatory cytokines are released following NLRP3 inflammasome activation and may further exasperate the inflammation [88].

### 3.4. The Progression of CSVD Heterogeneity Following Activation of NLRP3 Inflammasome

The magnitude of which the progression of certain CSVD manifestations (i.e., WMH, ePVs, etc.) may differ due to their exclusive etiopathogenesis (i.e., anatomical dependent) and socio-demographic factors has rarely been studied [89,90]. For example, deep subcortical WMHs may be due to axonal loss and/or arterio- or arteriolosclerosis, whereas periventricular WMHs mainly result from demyelination [90]. Moreover, ePVs in the basal ganglia may signify hypertensive arteriopathy, however lobar ePVS may signify CAA [89]. Hence, identification of the shared etiopathogenesis of CSVD manifestation is crucial when studying the progression of CSVD heterogeneity.

Recent studies have reported that some CSVD manifestations share exclusively similar pathogenesis, for example, CAA may cause the accumulation of amyloid (i.e., amyloid plaques) in the cerebral vasculature that supplies cortical and subcortical white matter. Blockage at the cortical vasculature may cause impairment in the cortical interstitial fluid drainage pathway or failure of waste clearance (i.e., fluidopathy or glymphatic system aberration), hence resulting in ePVS where an increased plaque in the subcortical region may result in WMHs [91,92]. Moreover, increased BBB permeability (due to endothelial dysfunction, results in plasma fluid components leakage) has been reported to be interlinked with CSVD manifestations such as WMHs with lacunes [93]. Taken together, cerebral microvascular lesions of ischemic origins (i.e., WMH, PVS, and lacunes) may promote neurodegenerative progression.

Furthermore, recent reports revealed that the NLRP3 inflammasome-mediated epithelial pyroptosis (i.e., mode of programmed cell death which is distinguished by the formation of plasma membrane pores mediated by inflammatory cytokines and caspase-1 release and plasma fluid components leakage) may accelerate the development of athero-, arterio-, and arteriolosclerotic plaques and increase plaque size [94,95], potentially influencing CSVD progression (see Figure 3). However, reports have also shown that the inhibition of NLRP3 inflammasome-mediated pyroptosis may possess neuroprotective benefits following cerebral ischemia [86,96] and potentially halting the progression of CSVD. Mechanistically, NLRP3 inflammasome-mediated pyroptosis is activated when caspase-1 cleaves GSDMD into C-terminal and N-terminal fragments. The N-terminal of GSDMD mediates cell lysis and the NLRP3 inflammasome activation [97]. Studies have shown that the N-terminal of the GSDMD lodges into the phospholipid bilayer by interacting with inner membrane glycerophospholipids such as phosphatidylserine, phosphatidylinositol phosphates, and phosphatidic acid, which in turn mediate the formation of pores (~20 nm) that lead to cellular blebbing and finally bursting, which releases inflammatory cytokines (i.e., IL-18 and IL-1β) [97,98] and circulating microparticles [99]. Circulating microparticles will then in turn heighten microvascular thrombosis [100].

Therefore, the NLRP3 inflammasome may promote cellular and neuronal injury or death arguably through pyroptosis and the formation of pro-thrombotic circulating microparticles. Furthermore, NLRP3 inflammasome-mediated pyroptosis is linked to NF-κB, with NF-κB possibly promoting *nlrp3* gene expression [17] and acting as a mediator for NLRP3 inflammasome-mediated pyroptosis to amplify the underlying heterogeneous manifestations of CVSD, including that of covert lesions.

### 3.5. The NLRP3 Inflammasome Inhibition as Neuroprotective Potential for CSVD

Multiple signaling pathways have been reported with regards to the onset and progression of cardio-cerebrovascular disease. However, limited studies address the signaling pathway for CSVD. Moreover, various drug studies have reported that the aggravation of the upstream NLRP3 inflammasome signaling pathway and IL-1β may serve as a neuroprotective potential for cerebral ischemia and CSVD. Several clinically available treatments for NLRP3 inflammasome-mediated diseases have been proposed, including drugs that target IL-1β (such as canakinumab—the neutralizing IL-1β antibody, anakinra—recombinant IL-1β receptor antagonist, and lilonapil—soluble decoy IL-1β receptor) [101,102]. Nonetheless, drugs that suppress the expression of the NLRP3 inflammasome are thought to be more economically efficient. Table 1 summarizes the current evidence on the available NLRP3 inflammasome inhibitors that can act as potential neuroprotection for CSVD.

Apart from the various drugs available, several innate signaling pathways that can reduce the onset and progression of CSVD have been identified. For example, the involvement of the IFN-β–NLRP3 signaling pathway. IFN-β acts as an anti-inflammatory agent in cerebrovascular disease by inhibiting NLRP3 inflammasome activity [119]. Furthermore, a recent study discovered that IFN-β can reduce the degradation of tight junction proteins in cerebral endothelial cells, thereby preventing the progression of CSVD to ischemic stroke [120]. In this case, IFN-β suppresses the production of ROS and induces the expression of anti-inflammatory agents such as IL-10 via a signal transducer and activator of transcription 1 (STAT1) dependent manner. Hence, IL-10 mitigates the production of pro-IL-1β [119,121] and subsequently inhibits the NLRP3 inflammasome. Besides that, STAT1 has been widely studied in cerebrovascular disease as it is associated with neuronal death. Therefore, the inhibition of STAT-1 and the NLRP3 inflammasome may play a neuroprotective role in CSVD and ischemic stroke.

Nonetheless, it is important to note that these signaling pathways may or may not correspond exclusively with the activation or suppression of NLRP3. The attempt to accurately inhibit or induce changes in these signaling pathways (as described above) is quite a challenge, as inhibiting or inducing one pathway may affect others. Therefore, specific inhibitors or drugs (as tabulated in Table 1) may offer prevention and therapeutic strategies for CSVD by targeting the NLRP3 inflammasome and its components.

## 4. Conclusions

The NLRP3 inflammasome has evolved into one of the most important components of the cerebral immune system that offers plausible mechanistic roles in the natural history of CSVD heterogeneous manifestations specifically, and cerebrovascular disease in general. Neuro-thrombo-inflammation mediated by the NLRP3 inflammasome has been linked to the progression of CSVD as the NLRP3 inflammasome mediates the production of pro-inflammatory cytokines (i.e., IL-1β) that increase cellular infiltration and BBB permeability, which can aggravate ischemic inflammation. Several specific drugs or compounds may target specific NLRP3 inflammasome components and inhibit the activation and action of the NLRP3 inflammasome that could prove beneficial for neuroprotective strategies of CSVD and other cerebrovascular diseases.

## Figures and Tables

**Table 1:** Evidence on NLRP3 inflammasome inhibitors as potential neuroprotection for CSVD.

Target	Findings	Refs.
Ethanol extract of *Canna x generalis* rhizome	Inhibits the expression of *nlrp3* gene and ASC mRNA Cleaved caspase-1 proteins, hence, inhibit the activation of NLRP3 inflammasomes Helps up-regulate occluding and claudin-1 expression following cerebral ischemia	[82,103]
ER2.4 and ER2.7 from *Hibiscus noldeae*	Inhibits the expression of IL-1β and IL-6 Inhibits pro-caspase-1 expression hence aggravating NLRP3 inflammasome activation	[104]
Water extract of *Artemisia scoparia*	Inhibits NF-κB Inhibits extracellular signal-regulated kinase-mediated NLRP3 and IL-1β gene expression Inhibits caspase-1 and IL-1β cleavage	[105]
Baicalin	Reduces caspase-1 mRNA and *nlrp3* gene expression Impedes the progression of atherosclerosis in mouse atherosclerotic model	[106]
6-shogaol, 8-shogaol, and 10-gingerol from ginger plant	Inhibits ATP-mediated caspase-1 activation Inhibits lipopolysaccharide-mediated pro-IL-1β and NLRP3 expression	[107]
MCC950 (or CP-456773 or CRID3)	Targets NEK7 and NLRP3 Hampers the interaction between NEK7 and NLRP3 Inhibits NLRP3 inflammasome in the NACHT domain Positive effect in mouse models of various NLRP3-inflammasome-mediated diseases Reduces blood pressure in hypertensive mice model Alleviates the production of IL-1β and IL-18 and inhibits neutrophil infiltration in ischemic disease Reduces brain lesion volume, and ameliorates cognitive and neurological function by reducing leukocyte recruitment, microglial activation, and pro-inflammatory cytokine release	[94,101,108,109,110]
Ginsenoside, Rg3	Targets NEK7 Hampers the interaction between NEK7 with NLRP3, and ASC with NLRP3 Inhibits the ASC oligomerization, hence altering the NLRP3 inflammasome cascade Inhibits K^+^ ion outflow-independent activation of NLRP3 inflammasome Inhibits K^+^ ion outflow-dependent interaction of NLRP3-NEK7	[111]
Oridonin	Targets NEK-NLRP3 interaction Acts as an anti-inflammatory, anti-oxidative, anti-tumor, and neuroregulatory Inhibits the secretion of IL-1β, IL-6, TNF-α, and NF-κB	[112,113]
CY-09	Inhibits the ASC-NLRP3 interaction Inhibits NLPR3-mediated inflammation in vivo Inhibits NLRP3 inflammasome, hence reducing the neuronal pyroptosis following ischemia Inhibits neuronal loss and astrocytes activation	[101,114]
Dapansutrile or OLT1177	Inhibits ASC-NLRP3 and NLRP3-caspase-1 interaction, hence inhibits polymerization of NLRP3 inflammasome Ameliorates cognitive impairment in Alzheimer’s disease mouse model Inhibits microglial cells to release TNF-α	[115,116]
Tranilast	Bind to NACHT domain of NLRP3 and inhibits NLRP3-ASC interaction Inhibits the activation of NLRP3 inflammasome by blocking the assembly and oligomerization of NLRP3 Inhibits the activation of NF-κB induced by cytokine Alleviates neuronal apoptosis following ischemia in the rat model	[117]
Dehydrocostus Lactone	Targets ASC Inhibits NLRP3 inflammasome activation in human peripheral blood mononuclear cells Ameliorates inflammatory response in lipopolysaccharide-mediated inflammation in mice model Inhibits NLRP3-ASC interaction, K^+^ ion outflow, IL-1β, and NF-κB expression	[118]
Z-YVAD-FMK	Targets caspase-1 and acts as an irreversible inhibitor for caspase-1, hence inhibiting the release of IL-1β and IL-18 and blocking the neuronal necrosis	[67]

Notes: ASC, apoptosis-associated speck-like protein; ATP, adenosine triphosphate; CRID3, cytokine release inhibitory drug 3; IL-1β, interleukin 1β; K^+^, potassium ion; NACHT, nucleotide-binding and oligomerization domain; NEK7, NIMA related kinase 7; NF-κB, nuclear factor kappa-light-chain enhancer of activated B cells; NLRP3, nucleotide-binding and oligomerization (NOD) domain-like receptor (NLR) family pyrin domain (PYD)-containing 3; mRNA, messenger ribonucleic acid; T2DM, type-2 diabetes mellitus; TNF-α, tumor necrosis factor alpha; Z-YVAD-FMK, Benzyloxycarbonyl-Tyr-Val-Ala-Asp(OMe)-fluoromethylketone.

## Data Availability

Not applicable.
